# In search of different categories of abstract concepts: a fMRI adaptation study

**DOI:** 10.1038/s41598-021-02013-8

**Published:** 2021-11-19

**Authors:** Francesca Conca, Eleonora Catricalà, Matteo Canini, Alessandro Petrini, Gabriella Vigliocco, Stefano F. Cappa, Pasquale Anthony Della Rosa

**Affiliations:** 1 ICoN Cognitive Neuroscience Center, Institute for Advanced Studies, IUSS Pavia, Palazzo del Broletto, Piazza Vittoria 15, 27100 Pavia, Italy; 2 IRCCS Fondazione Istituto Neurologico Casimiro Mondino, Pavia, Italy; 3 Unit of Neuroradiology, IRCCS Ospedale San Raffaele, Milan, Italy; 4 Department of Informatics, Università degli Studi di Milano, Milan, Italy; 5 Department of Experimental Psychology, University College London, London, UK

**Keywords:** Cognitive control, Language

## Abstract

Concrete conceptual knowledge is supported by a distributed neural network representing different semantic features according to the neuroanatomy of sensory and motor systems. If and how this framework applies to abstract knowledge is currently debated. Here we investigated the specific brain correlates of different abstract categories. After a systematic a priori selection of brain regions involved in semantic cognition, i.e. responsible of, respectively, semantic representations and cognitive control, we used a fMRI-adaptation paradigm with a passive reading task, in order to modulate the neural response to abstract (emotions, cognitions, attitudes, human actions) and concrete (biological entities, artefacts) categories. Different portions of the left anterior temporal lobe responded selectively to abstract and concrete concepts. Emotions and attitudes adapted the left middle temporal gyrus, whereas concrete items adapted the left fusiform gyrus. Our results suggest that, similarly to concrete concepts, some categories of abstract knowledge have specific brain correlates corresponding to the prevalent semantic dimensions involved in their representation.

## Introduction

Abstract concepts have been traditionally considered as a unique domain. According to the Dual Coding Theory abstract concepts rely solely on verbal information^1,2^. The Context Availability Theory described abstract concepts as less readily associated to contextual cues compared to concrete ones^3^. Additional proposals suggest that abstract concepts are organized by means of associative relations^4^, or represented through metaphors derived from concrete knowledge^5^.

According to “embodied” theories, concrete concepts involve distributed neural networks coding perceptual and motor information, differently contributing in characterizing specific categories, e.g. animals, tools^6,7^. If and how this framework applies to abstract concepts is still debated. Although the characterization of different abstract categories is complex due to the presence of fuzzy inter-categorical demarcations^8^, recent proposals suggest that they may be grounded in the brain regions representing specific semantic dimensions^9,10^. The hypothesis is that, in analogy to concrete knowledge^9,11^, different types of semantic dimensions, e.g. emotional-, social-, cognitive-, quantity-related, may support specific categories of abstract representations^12–14^. Neuroimaging studies have provided some supporting evidence, suggesting that specific dimensions may characterize different categories of concepts on the basis of the engagement of the brain networks involved in affective^15^, social and numerical processing^16^ during various tasks involving abstract concepts. The neural correlates of social and quantity-related categories are consistent, involving, respectively, the anterior temporal lobe and the intraparietal sulcus^16,17^, whereas for emotion-related concepts the results are more complex. Activations were reported in inferior frontal, motor/premotor areas^18^, rostral cingulate cortex^15^, anterior^19,20^, and mid-posterior temporal gyri^21^, see for a review^22^.

In the current study, we aimed to investigate the neural correlates of different abstract categories. We specifically selected emotions, attitudes, human actions and cognitions, based on previous neuropsychological studies including the same categories and reporting a selective preservation of emotion concepts in Alzheimer Disease^9,23^.

In accordance with the recent framework proposing that the storage and processing of word meaning is underpinned by neural systems subserving both the representation of conceptual knowledge and the control of its access, use and manipulation^24^, our first aim is to apply a novel approach combining literature review and BrainMap database in order to select the brain regions supporting both these processes. It has been suggested indeed that abstract concepts have a high variability in meaning and appear in a broad range of contexts and situations^25,26^, leading to the assumption that abstract, compared to concrete concepts pose higher demands on control functions^27^, revealed, for instance, by a greater activation of the left inferior frontal gyrus^28^.

To this aim, we used a functional magnetic resonance adaptation (fMRI-A) paradigm, presenting words pairs belonging to different concrete and abstract categories in a passive reading task. fMRI-A allows the exploration of the functional properties of a neural population, making use of the property displayed by some neurons of reducing their response to a repeatedly presented stimulus^29,30^. The underlying assumption is that, if the brain area remains adapted to the second stimulus, this indicates that its neural population is coding the attributes shared between the two stimuli^31^. This method was largely and successfully used with concrete stimuli like pictures of animals and faces^32,33^, but, to the best of our knowledge, never with abstract concepts.

We expect that presenting two concrete exemplars of the same category, e.g. two biological entities, will lead to activation suppression within the ventral visual areas tuned to concrete words^28^, resulting in a reduced fMRI signal. If the neurons within the voxel are domain-sensitive, differences should emerge only when considering concrete, but not abstract categories. Abstract concepts should adapt regions generally activated by these stimuli, e.g., mid-superior anterior temporal and/or rostral cingulate cortex for emotion-related words^15,21^. The observation of specific adaptation effects for additional abstract categories could give new insights into the organization of abstract knowledge. Since we adopted a passive reading task without explicit control demands, we do not expect to find adaptation in control-related regions for abstract or concrete categories.

## Results

### Abstract and concrete domains effects

The participant random effect was not significant (Wald Z = 1.568, p = 0.117) and the amount of overall data variance due to between‐subjects variability, expressed as intraclass correlation coefficient, was 0.006 (0.6%), thereby indicating that the inter-subject variability did not affect the results.

The CONDITION × ROI interaction revealed a significant effect in two semantic-related regions, namely the Left Middle Temporal Gyrus (L-MTG, rostral Brodmann Area 21, MNI coordinates: − 53, 2, − 30) (mean difference = 1268.81, CI 23.887; 2513.733) and the Left Fusiform Gyrus (L FG, rostro-ventral Brodmann Area 20, MNI coordinates: − 33, − 16, − 32) (mean difference = 1430.57, CI 185.647; 2675.492). In the DOMAIN × ROI interaction, significant differences were reported between ABS and CNC domains in L-MTG (mean difference = 1403.28, CI 158.354; 2648.200) and L Fusiform Gyrus (mean difference = 1904.59, CI 659.664; 3149.510).

**Figure 1:**
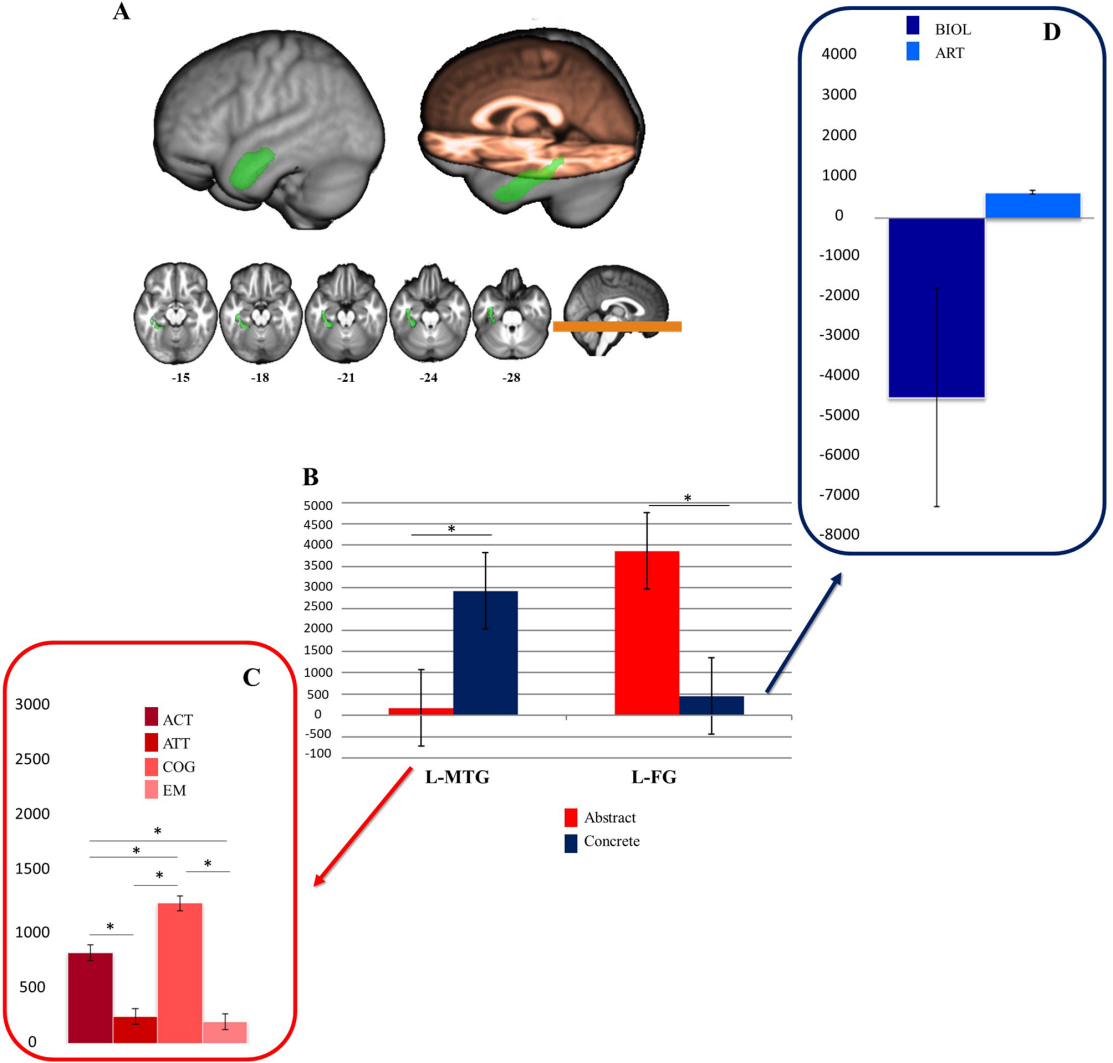
Anatomical location of L-MTG and L-FG (figure generated with MRIcron, v.1.0.20190902, https://www.nitrc.org/projects/mricron) (**A**), and plots with the mean eigenvariate values of Same Category–Same Word, for abstract and concrete domains (**B**) of abstract categories in L-MTG (**C**) and concrete categories in L-FG (**D**). Error bars indicate standard errors; *significant, bonferroni-corrected. See text for details. *L-MTG* left middle temporal gyrus, *L-FG* left fusiform gyrus, *BIOL* biological entities, *ART* artefacts, *ACT* human actions, *ATT* attitudes, *COG* cognitions, *EM* emotions.

In the three-way CONDITION × DOMAIN × ROI interaction, significant effects were found for the Same Category condition between the ABS and CNC domain in L-MTG and L-FG. In L-MTG the BOLD signal increase from the adaptation baseline (i.e. Same Word Condition) was significantly lower in the ABS domain (mean difference =  − 2754.63, CI − 4515.214; − 994.040), while in the L-FG the BOLD signal increase from the adaptation baseline (i.e. Same Word Condition) was significantly lower in the CNC domain (mean difference =  − 3407.68, CI − 5169.271; − 1647.098). This suggested the presence of adaptation effects in L-MTG and in L-FG for abstract and concrete domains, respectively. No comparable effects were found in the Different Category condition (see Fig. 1A,B, Supplementary Table S1 for the results in all the ROIs).

### Abstract and concrete categories effects

Next, we explored the dissociation found in the Same Category condition between abstract and concrete domains testing whether differences existed between categories for the abstract domain in L-MTG, and for the concrete domain in the L-FG, running, respectively, an ANOVA and a t-test on the BOLD contrast eigenvariate values (Same Category-Same Word).

For the ABS domain in L-MTG, we found a main effect of Category (F(3) = 109.366; p < 0.001). Post hoc Bonferroni-corrected tests revealed that emotions and attitudes did not differ between each other (mean difference = 49.078, p = 1, Cohen’s d = 0.39), but they significantly differ from cognitions (EM: mean difference =  − 1082.339, p < 0.001, Cohen’s d = 5.25; ATT: mean difference =  − 1033.261, p < 0.001, Cohen’s d = 5.19) and human actions (EM: mean difference =  − 630.369, p < 0.001, Cohen’s d = 2.88; ATT: mean difference =  − 581.292, p < 0.001, Cohen’s d = 2.74); cognition showed the greatest difference compared to all abstract categories (all p < 0.001). Specifically, whereas emotions and attitudes displayed the lowest BOLD signal increase from the adaptation baseline, i.e. index of adaptation effects, cognitions showed the greatest difference (Fig. 1C).

For the CNC domain in L-FG the difference between BOLD eigenvariate values of biological compared to artefacts categories was non significant (t(35) = − 1.882; p = 0.068, Cohen’s d = 0.44) (Fig. 1D).

For explorative purposes, we also performed a whole brain analysis evaluating the adaptation effect as higher activation in Different category compared to Same Category conditions, separately for each category (i.e. ACT, ATT, COG, EM, ART, BIOL). Results are shown in Supplementary Materials, Table S2.

## Discussion

We selected 15 semantic representation- and 11 semantic control-related regions from the integration of information derived from literature and BrainMap database, and used them to investigate the neural correlates of different kinds of abstract and concrete concepts, by means of an fMRI-A paradigm.

We found that the neural response associated to abstract and concrete concepts significantly differed in two semantic representation regions of the left anterior temporal lobe (ATL). The presentation of two concrete exemplars of biological entities and artefacts categories adapted the rostral L-FG, whereas two abstract exemplars of emotions and attitudes categories adapted the anterior L-MTG.

We showed distinct neural correlates for different semantic categories. Anterior fusiform gyrus was adapted by the concrete domain, with a qualitatively greater effect for biological entities (e.g., apple, zebra) than for artefacts (e.g., knife, airplane). This result confirms a role of the anterior fusiform in representing concrete in comparison to abstract concepts^34^, given its contribution in high level visual features processing^28^, including the retrieval of colour^35^, verification of physical properties^36^, and mental generation of features^37^. The repetition of semantically-related biological exemplars led to negative BOLD values compared to repeating the same word, inducing a stronger adaptation effect in comparison to artefacts, similar to the findings of Ref.^38^ in frontal, occipital and postcentral areas. This result is compatible with the role of the anterior fusiform gyrus in differentiating an item from similar competitors sharing visual and semantic features, specifically in the case of biological items^34,39^. Biological entities share indeed numerous highly correlated common properties, including shape and parts^40^.

In the abstract domain, we found a selective adaptation in the left aMTG for emotions (e.g., fear, happiness) and attitudes (e.g., dishonesty, tolerance). It is important to note that the categorization in the abstract domain is less definite from that of the concrete one. Lacking a taxonomic hierarchical organization, there are not clear boundaries between different classes, leading to a heterogeneity of the category composition, and an inconsistency in the label used to indicate similar concepts. For example, emotional concepts have been defined in the studies of Refs.^41,42^ as a third category, besides abstract and concrete, characterized by higher imageability and lower concreteness compared to other abstract words, and by lower imageability than concrete concepts. Accordingly, the emergence of two separate clusters for emotional and attitude concepts was reported in Ref.^13^, despite identified with different labels, i.e. emotive/inner states (e.g., anger) and self-sociality (e.g., politeness), respectively. However, the boundaries of these two categories are fuzzy, as emotions and attitudes have also many common features, for example they have been grouped together in a social-affective or endogenous factor by other studies using dimension ratings^43,44^.

This latter aspect is reflected by our result of a common neural substrate for both emotional and attitude concepts, with adaptation reported in the left aMTG for both. Accordingly, activations of left aMTG have been found during the retrieval of information regarding individuals, e.g., name, face identity, occupation, personality traits^45^, the attribution of adjectives defining people to a person name, using words close to ours (e.g., assertive)^46^, as well as in the representation of emotional-valenced social pictures and words^47^, and of emotion concepts^19,21^. The left aMTG, in some cases extending to the superior sections, is thought to contribute to socio-emotional contents, which may constitute important dimensions in representing the meaning of both attitudes- and emotions-related abstract concepts.

Finally, there are additional aspects characterizing emotional concepts, highlighting the heterogeneity of this class of concepts. Emotions have been described in fact in terms of valence (e.g. as in Ref.^15^, emotional experience^48^ and emotion-relatedness^49^, broadly referring to two main types: emotion referring terms (e.g. guilt, disdain) and emotion features terms (e.g. emergency, disease). To avoid the heterogeneity arising from the inclusion of both types of terms, in the current study we focused only on emotion referring words. Previous fMRI studies investigating only emotion-referring words in passive reading^18,49^ and word-typicality judgement tasks^50,51^ found an involvement of multiple regions, encompassing inferior frontal and precentral gyrus^49–51^, motor and premotor cortices^18^, and temporal lobe, extending from mid-posterior sections^49–51^, to the temporal pole^49–51^.

No adaptation effects were instead reported in the left aMTG for human actions (e.g., authorisation, punishment) and cognitions (e.g., mystery, logic), abstract categories less investigated. Concepts associated to cognition include words like *dream*, *reason*, *intellect*, and have been described as “referring to mental activity, ideas, opinions and judgements”^43,44^, involving orbitofrontal cortex^52^, face-related motor area^18^, and angular gyrus^19^. Both categories may include heterogeneous concepts, composed by different dimensions, probably preventing the involvement of specific regions^17^. Accordingly, on the basis of feature listing studies, cognitions have been characterized by a greater variability than other abstract concepts, eliciting information linked to the different events and situations in which they can occur^53^.

Notably, in the exploratory whole brain analysis, additional regions were reported to be involved in the processing of the different categories, e.g., supplementary motor areas, precentral gyri, cingulate cortex, middle and inferior temporal gyri, thus suggesting more widespread networks involved for the majority of abstract concepts.

These results posit important issues to the hub-and-spoke model, which suggests a graded specialization of ATL according to the differential connections to sensory, motor and limbic regions^24^. The superior and ventromedial ATL have been mostly involved, respectively, in processing abstract and concrete concepts, given their differential association to auditory/verbal and visual areas^27^, despite the data suggesting a specialization of superior ATL for social concepts has weakened its proposed role for all abstract concepts^54^. The selectivity of the middle ATL is less clear, since this region responded equally to verbal and visual inputs and to abstract and concrete concepts^55^. The ventro-lateral ATL, corresponding to anterior fusiform gyrus, has been instead considered the heteromodal representational hub, where all information converges, representing all types of concepts equally^56^. Crucially, our results suggest a stringent specialization of ATL, as only two categories of abstract concepts adapted the middle ATL, without any adaptation in the superior portion. Additionally, the anterior fusiform gyrus was selectively tuned for concrete and not for abstract concepts, in contrast with its role as semantic hub^56^.

Accordingly, previous studies suggested that not all abstract categories involved the anterior temporal lobe. A TMS experiment claimed a role of the intraparietal sulcus in the representation of quantity-related abstract concepts, e.g. immensity^16^, a finding which is further supported by behavioural data suggesting an impaired processing of these concepts in a patient with Cortico-Basal Syndrome, affecting the parietal lobes, but not in a patient with the semantic variant of primary progressive aphasia^10^.

Taken together, these data suggest the need to comprehensively include further different categories of abstract concepts and to consequently extend the focus of interest to large-scale brain networks, not restricted to the anterior temporal lobes.

Finally, we found no difference in adaptation in control-related regions between concrete or abstract concepts. Previous findings of a greater involvement of control-related areas for abstract compared to concrete concepts emerged from a variety of tasks, including lexical decision^8,57^, recognition memory^58^, synonym^27^ and semantic similarity judgement tasks^59^. All these tasks can be expected to engage control demands to a different degree, contrary to our task with minimal processing requirements.

In conclusion, our results are in line with the framework positing a cerebral distribution of semantic dimensions characterizing different categories of abstract concepts according to their content. While the present study was limited to nouns, conceptual differences, for example between emotional and mental states, are prominent also in the case of abstract verbs^60,61^, and needs to be further investigated. Additional studies are also needed to explore additional abstract dimensions/categories, including for example morality^12^ or theoretical^62^ information, as well as their interactions.

## Materials and methods

### Participants

36 healthy right-handed Italian subjects (mean age = 21.3 ± 2.5 years; 12 males) with normal hearing and vision, no history of neurological or psychiatric illness, and no early exposure to a second language participated. All provided written informed consent. The study complied with all provisions of the Declaration of Helsinki and was approved by the San Raffaele Hospital Ethics Committee.

### Stimuli

Ninety-six abstract and 96 concrete nouns were selected from Ref.^63^, following the categorization based on MultiWordNet database (http://multiwordnet.fbk.eu). First developed in English and then extended to other languages, MultiWordNet is endowed with a hierarchical organization, with words organized into a taxonomy and grouped together according to their meanings.

Selected abstract nouns belonged to 4 categories: emotions (EM), cognitions (COG), attitudes (ATT), human actions (ACT) according to MultiWordNet Domain and number of senses in MultiWordNet, including 24 stimuli for each category. Of note, the included nouns belonging to the emotion category indicated emotion-referring words. This choice prevented the possible heterogeneity often arising in including both emotion-referring (e.g. sadness) and emotion-features (e.g. emergency) words in the same set of stimuli^22^. Concrete nouns belonged to biological entities (BIOL) (e.g., animals, vegetables) and artefacts (ART) (e.g., items of furniture, tools), including 48 stimuli each. See Tables S3 and S4 in Supplementary Materials for the list of stimuli and variables taken into account.

Within each domain, word pairs were created, with half of the stimuli (n = 48) used as a prime and half as a target (i.e., first and second word in the pair). Prime and target were never switched in terms of order of presentation within each pair and were matched between categories, considering separately abstract (all p-values > 0.055) and concrete domains (all p-values > 0.056), for concreteness, imageability, familiarity, age of acquisition, context availability, abstractness, mode of acquisition (MoA), number of letters, written and spoken frequency (respectively from COLFIS: http://www.ge.ilc.cnr.it; and BADIP: http://badip.uni-graz.at/it/), distance from the median value of MoA of the specific category, frequency of senses in WordNet, number of senses and N-Synset in MultiWordNet.

For each domain, prime and target were combined into two conditions, Same Category, e.g. BIOL-BIOL or ACT-ACT, and Different Category, e.g. BIOL-ART or EM-ACT. Abstract and concrete nouns were never combined in a pair. This decision was in accordance with our main interest to focus on the different categories within abstract and concrete domains, specifically to explore whether different categories induced specific adaptation in relatively segregated brain areas.

To account for the semantic relatedness of the word pairs, Gloss Vector measure was considered. It combines the structure and content of WordNet with co–occurrence information derived from raw text and determines the relatedness of two concepts as the cosine of the angle between their gloss vectors^64^. For abstract and concrete domains, pairs in the Same Category condition were equally related (p at least 0.967), while pairs of the Different Category condition were equally unrelated (p = 0.1).

The number of pairs for each condition and the respective combinations, and the number of lists, pairs for each list and participants to whom the lists were administered are described in Supplementary Materials.

Twelve nouns (6 abstract; 6 concrete), not included in the previous sample, formed a Same Word semantic adaptation baseline condition, in which the same word was displayed as prime and target (e.g., Italian: sole-SOLE, English: sun-SUN, see Supplementary Materials, Table S6). This condition was introduced in order to tease apart effects related to repetition of semantic information from perceptual information related to the word form^38^. See Supplementary Materials for the variables considered for matching the stimuli between Same Word and Same Category conditions.

As repeated stimuli usually share also low-level visual properties, such as shape and orientation, we reduced stimulus durations to minimize the effects derived from early visual cortices^65^, and displayed prime and target respectively in lower and upper case, to minimize their perceptual similarity^66^.

The presence of semantic adaptation was measured by comparing the activation elicited by words belonging to the same category to the effect induced by the repetition of the same word.

### Passive reading task

**Figure 2:**
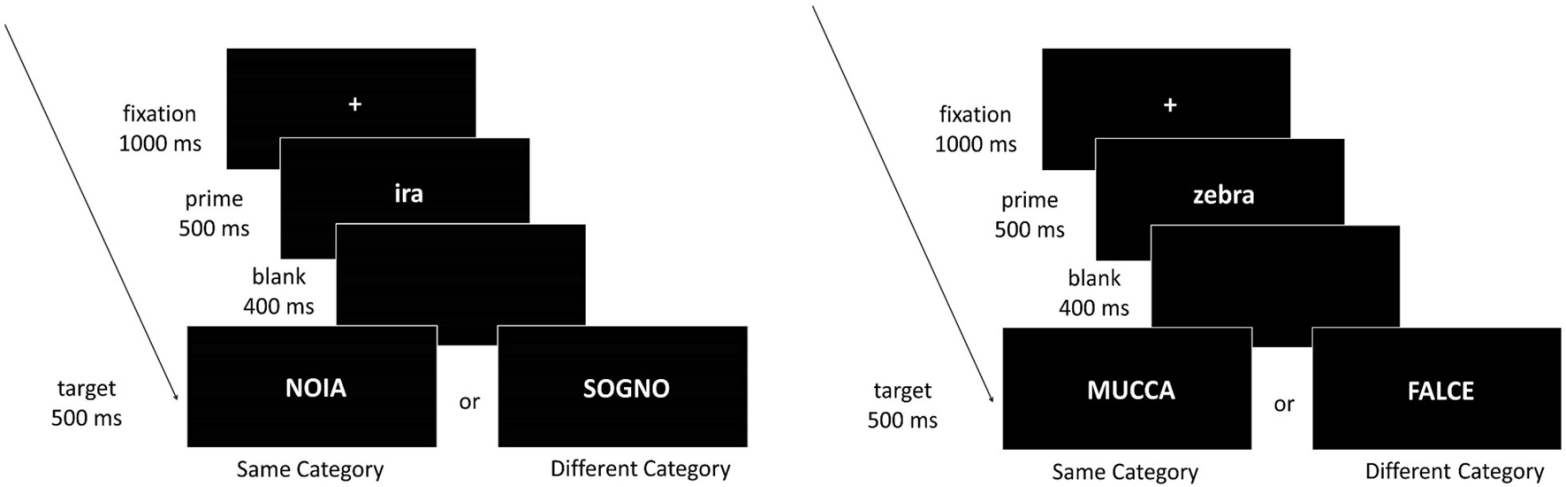
Timeline of an experimental trial, for abstract (left: Same Category condition: anger-BOREDOM; Different Category condition: anger-DREAM) and concrete (right: Same Category condition: zebra-COW; Different Category condition: zebra-SICKLE) domains. See text for details.

The task was a passive reading task where subjects were presented with abstract or concrete word pairs, consisting of two words belonging to the Same or Different Category conditions. On each trial a fixcross was presented for 1 s preceding the prime written in lower case (500 ms), followed by a blank screen (400 ms), and a target written in upper case (500 ms) (Fig. 2). Each trial was followed by a 3, 5 or 7 s jittered inter-trial interval (mean = 5.021 ms)^67^.

The experiment was divided into two runs, each one including 54 trials: 24 Same Category, 24 Different Category and 6 Same Word pairs, half abstract and half concrete.

Stimuli were presented with Presentation software (NeuroBehavioral Systems Inc.) via a PC outside the scanner room and delivered on a translucent screen at the foot of the magnet bore. Participants viewed the screen through a mirror system attached to the top of the head coil. They were instructed to silently read the words without moving their lips or tongue.

Prior to fMRI scanning, participants read the instructions and performed a training session, consisting in 10 trials not included in the experiment to familiarize with the task.

### fMRI data acquisition

An fMRI event-related technique was adopted (3 T Intera Philips body scanner, Philips Medical Systems, Best, NL, 8 channels-sense head coil, sense reduction factor = 2, TE = 30 ms, TR = 2000 ms, FOV = 240 × 240, matrix size = 96 × 96, 38 axial slices per volume, 191 volumes for each run, slice thickness = 3 mm).

Optimal EPI parameters at 3 T were chosen to gain BOLD sensitivity in temporal and frontal regions^68^ and slice tilt was set to 20° on the (RL) tangent to minimize susceptibility induced artefacts and signal dropouts. The phase encoding gradient polarity was chosen to be negative with the phase encoding direction going from the anterior part to the posterior part of the brain^8^.

Each run was anticipated by five dummy scans, discarded before analysing the data to optimize EPI image signal. For each participant, a high-resolution structural image was acquired (MPRAGE, 150 slice T1-weighted image TR = 8.03 ms, TE = 4.1 ms; flip angle = 8°, TA = 4.8 min, resolution = 1 × 1 × 1 mm) in the axial plane for coregistration, segmentation, spatial normalization of the EPI scans.

### fMRI data preprocessing and analysis

Image preprocessing was performed using SPM8 (http://www.fil.ion.ucl.ac.uk/spm) following the procedures adopted in our previous work^8^. Data preprocessing for each subject included: (1) EPI time-series diagnostics using tsdiffana (Matthew Brett, MRC CBU, http://imaging.mri-cbu.cam.ac.uk/imaging/DataDiagnostics), (2) alignment and orientation of structural images to improve segmentation accuracy, (3) co-registration of EPI scans to the structural volume, (4) T1-weighted image tissue segmentation using the ‘new segment’ tool in SPM8 and generation of deskulled bias-corrected T1 images, (5) study-specific template creation using diffeomorphic image registration (DARTEL) in SPM8 and subject-specific flow fields generation containing the spatial deformations to normalize the EPI images into a common MNI coordinate space, (6) co-registered EPI time-series noise filtering (ArtRepair toolbox: http://cibsr.stanford.edu/tools/ArtRepair/ArtRepair.htm), motion and distortion correction using subject-specific field-map parameters (realign and unwarp) and suppression of residual motion effects with Art Repair toolbox, (7) creation of a deskulled mean functional mask to remove nonbrain tissue from co-registered, noise-, motion-, and distortion-corrected EPI time-series in order to increase sharpness and avoid mismatch between alignment of the EPI data to the T1 image, (8) affine normalization of EPI data to MNI space with DARTEL flow fields, according to smooth deformations for each subject’s native space gray, (9) spatial smoothing, with Gaussian kernel of 6 mm.

At the first single-subject level, the 10 experimental conditions were used as separate regressors according to 6 pseudo-randomized lists resulting from the combinations of Same and Different Category conditions in ABS and CNC domains, and Same Word conditions. The conditions were modelled by convolving a delta function of each event type with a “canonical” hemodynamic response as the basis function to create regressors of interest. Low frequency signal drifts were removed with a high-pass filter (128 s) and AR1 correction for serial autocorrelation was applied. Second level group analyses using participants as a random effect were performed on contrast images for Same Category minus Same Word and Different Category minus Same Word conditions for all categories in the ABS and CNC domains derived from 1st level analyses (n = 36 participants).

### Regions of interest selection

**Figure 3:**
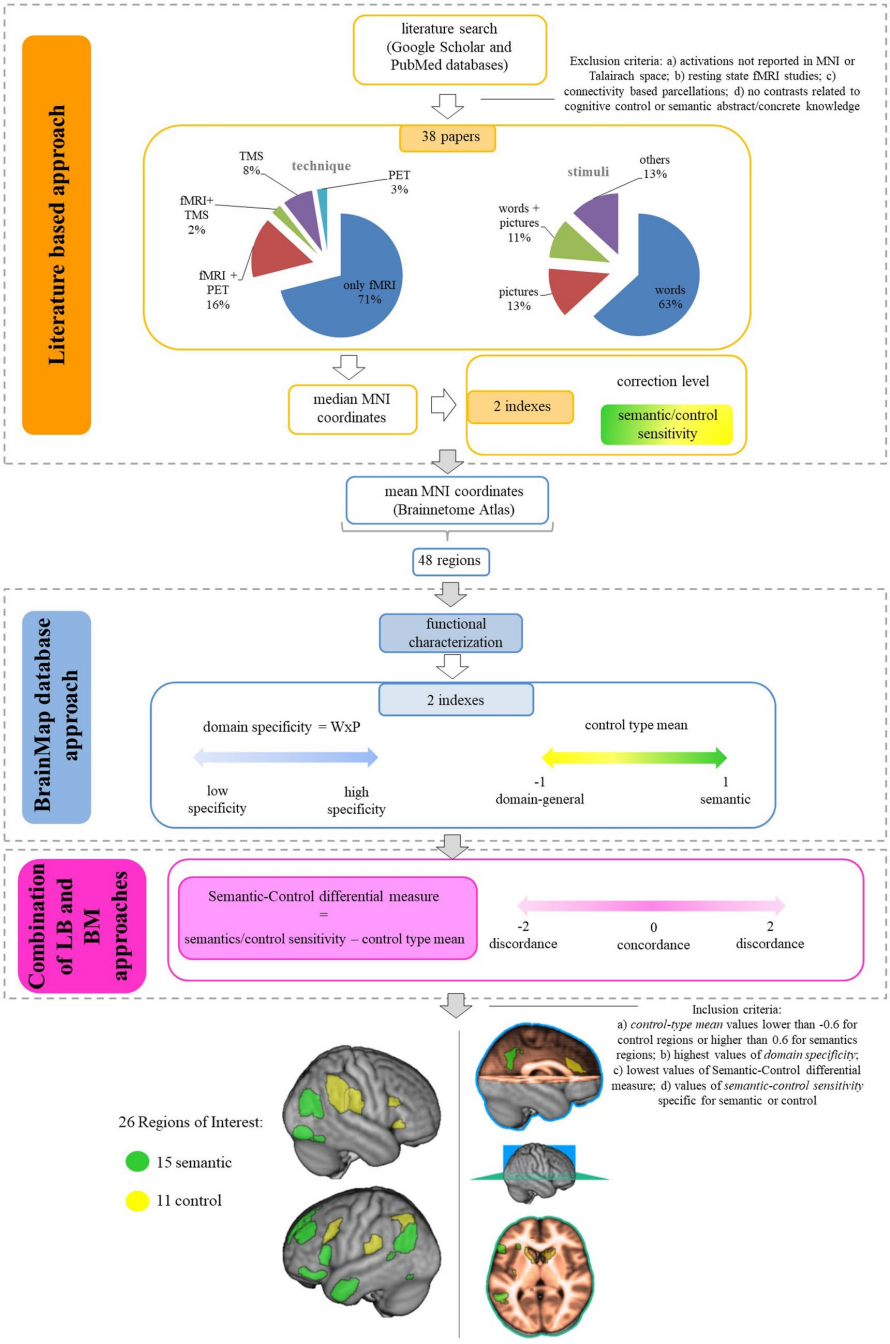
Schematic illustration of ROIs’ selection process.

We created a map of brain regions underlying semantic knowledge representation and cognitive control. We used two different approaches, based on literature (LB) and BrainMap database (BM), and their combination, to create five indexes for the selection of the ROIs, see Fig. 3.

#### Literature based approach

Our starting point was a literature search using Google Scholar and PubMed databases, selecting those studies investigating the domains of semantics (e.g., concrete and abstract domains of knowledge) and cognitive control. We used different combinations of the following terms, with both extended and abbreviated forms: semantic dimensions, semantic cognition, cognitive control, concrete/abstract dimensions/concepts/categories, social, emotions, mental states, living and non-living, functional Magnetic Resonance Imaging, Positron Emission Tomography, Transcranial Magnetic Stimulation, Electroencephalography, Magnetoencephalography. We additionally looked for relevant studies by manual search starting from the lists of references of the retrieved papers. Exclusion criteria were: (a) clusters of activations in brain regions not reported in MNI or Talairach reference space (coordinates in Talairach space were converted into MNI space using Tal2MNI function in Matlab), (b) resting state fMRI based studies, (c) connectivity-based brain parcellations, (d) studies not reporting specific contrasts related to cognitive control or semantic abstract/concrete domains of knowledge.

38 papers were included: 31 original research papers and 7 meta-analyses. fMRI was used in all included studies, alone (n = 27) or in combination with other methods (e.g. PET: n = 6; TMS: n = 1). Among the original research papers, the majority included healthy subjects (n = 30) and only one dealt with patients; the mean number of participants per study was 16.13 subjects (range 3–32). The stimuli used were only words (n = 24), only pictures (n = 5), both words and pictures (n = 4), and other types of stimuli (e.g., arrows in the Flanker task) (n = 5). See Table S7 in the Supplementary Materials for the list of the included studies.

For each region we calculated two indexes, a *correction level index* and *semantics/control sensitivity index.* The former index was aimed to individuate the regions resulting from the more stringent analyses, whereas the latter was motivated by the need to characterize each region as semantic or control-related, according to its prevailing involvement in one of the two aspects of semantic cognition.

##### Correction level index

For each paper, we considered only one contrast relative to semantics or control and one x, y, z coordinate per region. If activation cluster coordinates for two or more contrasts had a distance greater than 10 mms, they were included. Among the included contrasts (n = 195), most (n = 122) were thresholded at p-values corrected for multiple comparisons, including false discovery rate correction (n = 60), family-wise error correction (n = 28), or combined different methods for multiple-comparison correction (n = 34); 56 contrasts were uncorrected (n = 33 with p < 0.001; n = 10 with p < 0.005; n = 10 with p < 0.05; n = 3 with p < 0.01); and for 17 contrasts such information was not available. A mean value was calculated for multiple coordinates. For each region a single value ranging from − 1 to 1 was assigned and coded as *correction level index*. The level of correction for false-positives was coded as “1” for voxel-level corrections, “0.5” for cluster-level corrections and “ − 1” for uncorrected p-values.

##### Semantics/control sensitivity index

A mean value for each region ranging from − 1 (i.e. Control) to 1 (i.e. Semantics) was calculated and coded as *semantics/control sensitivity index*. Contrasts were coded as “1” for the well-known specific concrete semantic categories according to the previous literature (e.g., naming tools > naming animals); “0.5” for other possible semantic abstract categories, e.g., social, morality, characterized by fuzzier and more blurred boundaries (e.g., social > non-social words); and “− 1” for control (e.g., incongruent > congruent trials). For instance, a brain area with a corresponding *semantics/control sensitivity index* of − 1 displayed a high sensitivity for control over semantics and viceversa.

The median coordinate x-, y- and z-values resulting from the different contrasts for each region were then calculated. We mapped these median coordinates in the human Brainnetome Atlas (http://atlas.brainnetome.org)^69^, which includes 210 cortical and 36 subcortical brain areas, characterized in terms of connectivity, anatomical and cytoarchitectonic features. Median coordinates derived from different contrasts in different papers corresponding to the same region in the Atlas were collapsed, thus a total of 48 regions was considered for the mapping procedures.

#### BrainMap database approach

The second step of our procedure was to functionally characterize the 48 regions emerging from literature review by means of BrainMap database (http://www.brainmap.org/taxonomy). The aim of this characterization was twofold. On one hand, we used the information of the behavioral domains in order explore the specificity of the functional processes associated with the region, and, on the other hand, we used the data of paradigm classes, namely the types of experimental tasks, to characterize the region as semantic or control-related.

##### Domain specificity

Cognitive domains included the macro-domains of Action, Cognition, Emotion, Interoception and Perception, with possible micro-domains (e.g., Orthography; Phonology; Semantics; Speech; Syntax for language). For each domain, to individuate the heterogeneity of domains involved for each region, we calculated the value *W*, taking into account the number of both micro and macro-domains. The formula was as follows:

$$W= \frac{\frac{n. \,of\, micro-domains\, of\, the\, region}{total \,n.\, of\, micro-domains} + \frac{n. \,of\, macro-domains \,of\, the\, region }{total \,n.\, of \,macro-domains} }{total \,n. \,of \,domains\, of \,the\, region}.$$

We then computed the *domain specificity index*, as the product of the value *W* for the likelihood *P* of observing activations in a brain region given a specific cognitive domain (i.e., *Domain specificity* = *W* × *P*), for each domain of each brain region.

##### Control type mean

We categorized paradigms classifying the type of control involved in each (i.e. control-type), assigning the values of “1” for predominantly semantic paradigms (e.g., Semantic Monitor/Discrimination), “0.5” for mixed domain-general/language-specific paradigms (e.g., Phonological Discrimination), and “− 1” for control paradigms (e.g., Flanker Task). A *control-type mean* value for each region was then calculated.

#### Combination of LB and BM approaches

The last step of our procedure consisted in the combination of LB and BM information to select brain regions underlying, respectively, semantics or control processing. In order to evaluate the concordance between the two approaches we computed for each region the Semantic-Control differential measure (i.e., *[LB: semantics/control sensitivity index] − [BM: Control Type mean]*). A value of zero indicates a perfect concordance.

Regions were selected based on *control-type mean* values lower than − 0.6 to be included in control regions or higher than 0.6 for semantics regions, and had also to display highest values of *domain specificity*, lowest values of Semantic-Control differential measure, and values of *semantic-control sensitivity* specific for semantic or control. This procedure led to the inclusion of 15 semantic and 11 control regions for a total of 26 ROIs (see Fig. 3 below and Table S8 in Supplementary Materials).

### Analysis of BOLD signal

#### Extraction of BOLD signal

We used REX toolbox (https://www.nitrc.org/projects/rex/) to extract the BOLD signal from the 26 ROIs for (1) beta images relative to the 10 conditions of interest at the 1st subject-level, used for outliers values screening, and (2) contrast images (i.e. linear combination of beta images) coding comparisons between Same Category and Different Category conditions with Same Word condition at the 2nd group level, thus obtaining a subjects X regions matrix including BOLD signal estimates extracted for all betas relative to all conditions of interest at the 1st and 2nd level. For each region, we extracted the eigenvariate values (first component, corresponding to the values that summarized signal across voxels by means of Singular Value Decomposition), with a within-ROI scaling procedure.

No subjects or ROIs were identified as outliers (see Supplementary Materials for details).

#### ROIs data analyses

Analyses were performed with SPSS software (IBM SPSS Statistics 20) on the extracted eigenvariate values on BOLD estimates extracted from the 26 ROIs from contrast images testing for Same Category–Same Word and Different Category–Same Word differences. Lower differences of the BOLD signal in a ROI between the same category condition and the same word condition (i.e., the adaptation baseline), was taken as evidence of larger adaptation effects.

Our main aim was to unveil whether state-dependent effects (i.e., adaptation or enhancement^29^) were detectable at the semantic level net of the repetition of perceptual information related to word form, i.e. Same Word condition, and whether these effects were different for abstract and concrete domains.

We first entered contrast BOLD eigenvalues in a Linear Mixed Effect Model, which allows controlling for subject variability, with CONDITION (i.e., Same Category-Same Word, Different Category–Same Word), DOMAIN (i.e., ABS, CNC), and ROI (n = 26) (Table S8 in Supplementary Materials) as within-subjects factors. In order to account for the randomization of stimuli between participants, we included the participants as random factor in the model. The model was tested using repeated-measures analysis of variance (rANOVA). To explore possible two-way (i.e. CONDITION × ROI) or three-way interactions (i.e. CONDITION × DOMAIN × ROI) in specific ROIs, paired-sample t-tests or ANOVA models, Bonferroni-corrected, were used to compare contrast estimate means for significant ROIs.

## Supplementary Information

Supplementary Information.
