# Supplementary material for: In search of different categories of abstract concepts: a fMRI adaptation study
Source: Sci Rep. 2021 Nov 19;11:22587. doi: 10.1038/s41598-021-02013-8 (PMC8604982; doi:10.1038/s41598-021-02013-8)

**Supplementary materials**

**Table 1.** Results of the three-way CONDITION x DOMAIN x ROI interaction. * = significant effects, bonferroni-corrected.

|  |  |  | **Same Category- Same Word** | | | **Different Category- Same Word** | | |
| --- | --- | --- | --- | --- | --- | --- | --- | --- |
| *ROI* |  |  | *mean difference* | *95% Confidence Interval for Difference (lower bound)* | *95% Confidence Interval for Difference (upper bound)* | *mean difference* | *95% Confidence Interval for Difference (lower bound)* | *95% Confidence Interval for Difference (upper bound)* |
| SFG_L_7_3 | ABS | CNC | -22.786 | -1783.373 | 1737.801 | -40.655 | -1801.242 | 1719.932 |
|  | CNC | ABS | 22.786 | -1737.801 | 1783.373 | 40.655 | -1719.932 | 1801.242 |
| SFG_L_7_6 | ABS | CNC | -0.233 | -1760.820 | 1760.354 | -114.932 | -1875.519 | 1645.655 |
|  | CNC | ABS | 0.233 | -1760.354 | 1760.820 | 114.932 | -1645.655 | 1875.519 |
| MFG_L_7_1 | ABS | CNC | 298.686 | -1461.901 | 2059.273 | -234.317 | -1994.904 | 1526.270 |
|  | CNC | ABS | -298.686 | -2059.273 | 1461.901 | 234.317 | -1526.270 | 1994.904 |
| MFG_L_7_2 | ABS | CNC | -3.355 | -1763.941 | 1757.232 | 3.277 | -1757.310 | 1763.864 |
|  | CNC | ABS | 3.355 | -1757.232 | 1763.941 | -3.277 | -1763.864 | 1757.310 |
| IFG_R_6_1 | ABS | CNC | 2.253 | -1758.334 | 1762.840 | 3.09 | -1757.496 | 1763.677 |
|  | CNC | ABS | -2.253 | -1762.840 | 1758.334 | -3.09 | -1763.677 | 1757.496 |
| IFG_L_6_3 | ABS | CNC | 66.122 | -1694.465 | 1826.709 | -5.995 | -1766.582 | 1754.592 |
|  | CNC | ABS | -66.122 | -1826.709 | 1694.465 | 5.995 | -1754.592 | 1766.582 |
| IFG_R_6_5 | ABS | CNC | 210.594 | -1549.992 | 1971.181 | 58.042 | -1702.545 | 1818.629 |
|  | CNC | ABS | -210.594 | -1971.181 | 1549.992 | -58.042 | -1818.629 | 1702.545 |
| OrG_L_6_6 | ABS | CNC | 67.078 | -1693.508 | 1827.665 | 16.808 | -1743.778 | 1777.395 |
|  | CNC | ABS | -67.078 | -1827.665 | 1693.508 | -16.808 | -1777.395 | 1743.778 |
| MTG_L_4_2 | ABS | CNC | -2754.63* | -4515.214 | -994.040 | -51.927 | -1812.514 | 1708.660 |
|  | CNC | ABS | 2754.627* | 994.040 | 4515.214 | 51.927 | -1708.660 | 1812.514 |
| FuG_L_3_1 | ABS | CNC | 3407.684* | 1647.098 | 5168.271 | 401.489 | -1359.098 | 2162.076 |
|  | CNC | ABS | -3407.68* | -5168.271 | -1647.098 | -401.489 | -2162.076 | 1359.098 |
| FuG_R_3_2 | ABS | CNC | 37.17 | -1723.417 | 1797.757 | -323.383 | -2083.970 | 1437.204 |
|  | CNC | ABS | -37.17 | -1797.757 | 1723.417 | 323.383 | -1437.204 | 2083.970 |
| FuG_L_3_3 | ABS | CNC | 419.941 | -1340.646 | 2180.528 | -403.912 | -2164.499 | 1356.675 |
|  | CNC | ABS | -419.941 | -2180.528 | 1340.646 | 403.912 | -1356.675 | 2164.499 |
| FuG_R_3_3 | ABS | CNC | -40.831 | -1801.418 | 1719.756 | 180.978 | -1579.609 | 1941.565 |
|  | CNC | ABS | 40.831 | -1719.756 | 1801.418 | -180.978 | -1941.565 | 1579.609 |
| pSTS_L_2_2 | ABS | CNC | -29.169 | -1789.756 | 1731.418 | -17.303 | -1777.890 | 1743.284 |
|  | CNC | ABS | 29.169 | -1731.418 | 1789.756 | 17.303 | -1743.284 | 1777.890 |
| IPL_R_6_1 | ABS | CNC | 297.478 | -1463.109 | 2058.064 | -48.172 | -1808.759 | 1712.414 |
|  | CNC | ABS | -297.478 | -2058.064 | 1463.109 | 48.172 | -1712.414 | 1808.759 |
| IPL_L_6_2 | ABS | CNC | -4.755 | -1765.342 | 1755.832 | -3.96 | -1764.547 | 1756.627 |
|  | CNC | ABS | 4.755 | -1755.832 | 1765.342 | 3.96 | -1756.627 | 1764.547 |
| IPL_R_6_4 | ABS | CNC | -4.958 | -1765.545 | 1755.629 | 8.645 | -1751.942 | 1769.231 |
|  | CNC | ABS | 4.958 | -1755.629 | 1765.545 | -8.645 | -1769.231 | 1751.942 |
| IPL_L_6_5 | ABS | CNC | -40.492 | -1801.078 | 1720.095 | 69.074 | -1691.513 | 1829.661 |
|  | CNC | ABS | 40.492 | -1720.095 | 1801.078 | -69.074 | -1829.661 | 1691.513 |
| IPL_L_6_6 | ABS | CNC | -167.557 | -1928.144 | 1593.029 | -953.385 | -2713.971 | 807.202 |
|  | CNC | ABS | 167.557 | -1593.029 | 1928.144 | 953.385 | -807.202 | 2713.971 |
| IPL_R_6_6 | ABS | CNC | -236.99 | -1997.576 | 1523.597 | 20.236 | -1740.351 | 1780.822 |
|  | CNC | ABS | 236.99 | -1523.597 | 1997.576 | -20.236 | -1780.822 | 1740.351 |
| Pcun_R_4_4 | ABS | CNC | -18.801 | -1779.388 | 1741.786 | -3.09 | -1763.677 | 1757.497 |
|  | CNC | ABS | 18.801 | -1741.786 | 1779.388 | 3.09 | -1757.497 | 1763.677 |
| CG_L_7_3 | ABS | CNC | 21.512 | -1739.075 | 1782.099 | -111.517 | -1872.104 | 1649.069 |
|  | CNC | ABS | -21.512 | -1782.099 | 1739.075 | 111.517 | -1649.069 | 1872.104 |
| OcG_R_4_4 | ABS | CNC | -206.962 | -1967.549 | 1553.624 | 227.183 | -1533.404 | 1987.770 |
|  | CNC | ABS | 206.962 | -1553.624 | 1967.549 | -227.183 | -1987.770 | 1533.404 |
| Hipp_L_2_1 | ABS | CNC | 60.173 | -1700.414 | 1820.760 | 6.519 | -1754.067 | 1767.106 |
|  | CNC | ABS | -60.173 | -1820.760 | 1700.414 | -6.519 | -1767.106 | 1754.067 |
| Str_R_6_1 | ABS | CNC | -49.651 | -1810.237 | 1710.936 | 170.946 | -1589.641 | 1931.533 |
|  | CNC | ABS | 49.651 | -1710.936 | 1810.237 | -170.946 | -1931.533 | 1589.641 |
| Str_L_6_5 | ABS | CNC | -1507.98 | -3268.568 | 252.605 | 78.113 | -1682.474 | 1838.700 |
|  | CNC | ABS | 1507.982 | -252.605 | 3268.568 | -78.113 | -1838.700 | 1682.474 |

**Table 2.** Regions showing adaptation effect evaluated as Different Category vs Same Category conditions by means of independent t-test (for EM, ATT, ACT, COG categories) and paired-sample t-tests (ART, BIOL categories), in a whole brain analysis. Effects are displayed at an uncorrected p value < 0.001, extent threshold= 3 voxels. L= left; R= right; AAL= Automatic Anatomical Labelling.

| voxels | Z | p-value uncorrected | MNI coordinates, mm | | | Brain Region (AAL) |
| --- | --- | --- | --- | --- | --- | --- |
|  |  |  | X | Y | Z |  |
| adaptation ACT | | | | | | |
| 5 | 4.13 | <0.001 | -10 | 18 | 54 | Supp Motor Area L |
| 4 | 3.5 | <0.001 | -25 | -73 | 6 | Calcarine L |
| adaptation ATT | | | | | | |
| 47 | 4.2 | <0.001 | 18 | -30 | 63 | Precentral R |
| 19 | 3.77 | <0.001 | -15 | -33 | 69 | Paracentral Lobule L |
| 17 | 4.68 | <0.001 | 10 | -20 | 12 | Thalamus R |
| 16 | 3.82 | <0.001 | -5 | -10 | 66 | Supp Motor Area L |
| 12 | 4.08 | <0.001 | 10 | -10 | 57 | Supp Motor Area R |
| 11 | 3.96 | <0.001 | 43 | -55 | -3 | Temp Inf R |
| 11 | 3.45 | <0.001 | 48 | -25 | 57 | Postcentral R |
| 9 | 3.6 | <0.001 | -5 | 13 | 48 | Supp Motor Area L |
| 7 | 3.78 | <0.001 | -5 | -28 | 63 | Paracentral Lobule L |
| 6 | 3.64 | <0.001 | 18 | -63 | 0 | Lingual R |
| 5 | 3.83 | <0.001 | -20 | -50 | -30 | Cerebelum L |
| 4 | 3.68 | <0.001 | 38 | -50 | 60 | Parietal Sup L |
| 4 | 3.54 | <0.001 | 3 | -23 | 66 | Supp Motor Area R |
| 4 | 3.35 | <0.001 | -25 | -78 | 21 | Occipital Mid L |
| 3 | 3.37 | <0.001 | -8 | -10 | 45 | Cingulum Mid L |
| 3 | 3.28 | 0.001 | -53 | -15 | 45 | Postcentral L |
| 3 | 3.28 | 0.001 | -33 | 25 | 3 | Insula L |
| adaptation COG | | | | | | |
| 9 | 3.57 | <0.001 | 25 | -33 | 15 | Hippocampus R |
| 5 | 3.73 | <0.001 | 35 | -8 | -21 | Hippocampus R |
| 3 | 3.72 | <0.001 | -18 | -5 | 27 | Caudate L |
| 3 | 3.43 | <0.001 | -30 | -40 | 15 | Rolandic oper L |
| 3 | 3.43 | <0.001 | -18 | 38 | 15 | Cingulum Ant L |
| 3 | 3.38 | <0.001 | 3 | -30 | 21 | Cingulum Post L |
| 3 | 3.36 | <0.001 | -18 | -35 | 24 | Caudate L |
| 3 | 3.19 | 0.001 | 20 | -65 | 27 | Cuneus R |
| adaptation EM | | | | | | |
| 7 | 3.84 | <0.001 | 20 | 30 | 6 | Caudate R |
| 7 | 3.64 | <0.001 | 10 | 13 | 18 | Caudate R |
| 5 | 3.41 | <0.001 | 48 | -68 | 9 | Temporal Mid R |
| 4 | 3.31 | <0.001 | -8 | 0 | 24 | Caudate L |
| 3 | 3.24 | 0.001 | -8 | 28 | 6 | Caudate L |
| adaptation BIOL | | | | | | |
| 3 | 3.56 | <0.001 | -10 | 15 | 18 | Caudate L |
| adaptation ART | | | | | | |
| 12 | 3.94 | <0.001 | -13 | 20 | 51 | Frontal Sup L |
| 4 | 3.69 | <0.001 | 33 | -18 | 54 | Precentral R |

**Table 3.** List of the experimental stimuli, separately for abstract (ABS) and concrete (CNC) category.

| **ABS category** | **prime (Italian)** | **prime (English)** | **target (Italian)** | **target (English)** |
| --- | --- | --- | --- | --- |
| Human Actions (ACT) | abbandono | neglect | difesa | defense |
|  | approvazione | authorisation | educazione | education |
|  | assoluzione | absolution | fallimento | default |
|  | carriera | career | inganno | deception |
|  | conflitto | conflict | peccato | sin |
|  | dovere | duty | preghiera | pray |
|  | preparazione | preparation | responsabilità | responsibility |
|  | punizione | punishment | rimedio | remedy |
|  | riduzione | reduction | scoperta | discovery |
|  | salvezza | salvation | seduzione | seduction |
|  | scherzo | joke | tradimento | betrayal |
|  | torto | wrong | vendetta | revenge |
| Attitudes (ATT) | avarizia | avarice | bellezza | beauty |
|  | differenza | difference | calma | quietness |
|  | disonestà | dishonesty | fascino | charm |
|  | follia | madness | gentilezza | kindness |
|  | forza | strength | importanza | importance |
|  | giustizia | justice | innocenza | innocence |
|  | immortalità | immortality | lealtà | loyalty |
|  | insolenza | insolence | onore | honour |
|  | insufficienza | deficiency | originalità | originality |
|  | mediocrità | mediocrity | pazienza | patience |
|  | merito | merit | stile | style |
|  | virtù | virtue | tolleranza | tolerance |
| Cognitions (COG) | concetto | concept | curiosità | curiosity |
|  | dimenticanza | forgetfulness | fantasma | ghost |
|  | esitazione | hesitation | fiducia | trust |
|  | filosofia | philosophy | illusione | illusion |
|  | giudizio | judgement | inesperienza | inexperience |
|  | ideale | ideal | invenzione | creation |
|  | incertezza | uncertanty | istinto | instinct |
|  | incredulità | disbelief | mistero | mystery |
|  | logica | logic | scopo | purpose |
|  | principio | principle | sogno | dream |
|  | saggezza | wisdom | sospetto | suspicion |
|  | schema | scheme | talento | talent |
| Emotions (EM) | disprezzo | disdain | agonia | agony |
|  | fervore | fervour | amarezza | bitterness |
|  | furia | rage | amore | love |
|  | gioia | happiness | entusiasmo | eagerness |
|  | inquietudine | inquietude | esasperazione | exasperation |
|  | ira | anger | invidia | envy |
|  | panico | panic | noia | boredom |
|  | paura | fear | odio | hate |
|  | risentimento | resentment | passione | passion |
|  | simpatia | sympathy | soddisfazione | satisfaction |
|  | sollievo | relief | tristezza | sadness |
|  | tormento | anguish | vergogna | shame |

| **CNC category** | **prime (Italian)** | **prime (English)** | **target (Italian)** | **target (English)** |
| --- | --- | --- | --- | --- |
| Biological Entities (BIOL) | alghe | seaweed | albicocca | apricot |
|  | asino | donkey | arancia | orange |
|  | asparago | asparagus | banana | banana |
|  | braccio | arm | cammello | camel |
|  | cane | dog | carota | carrot |
|  | carciofo | artichoke | cavallo | horse |
|  | ciliegie | cherries | cuore | heart |
|  | cipolla | onion | elefante | elephant |
|  | gallina | hen | erba | grass |
|  | gallo | rooster | fragola | strawberry |
|  | giraffa | giraffe | fungo | mushroom |
|  | insalata | salad | gufo | owl |
|  | limone | lemon | insetto | bug |
|  | naso | nose | leone | lion |
|  | noce | walnut | maiale | pig |
|  | oca | goose | mela | apple |
|  | palma | palm | melanzana | aubergine |
|  | pappagallo | parakeet | mucca | cow |
|  | pera | pear | muscolo | muscle |
|  | piccione | pigeon | orso | bear |
|  | pomodoro | tomato | pecora | sheep |
|  | topo | mouse | peperone | pepper |
|  | zebra | zebra | quercia | oak |
|  | zucca | pumpkin | uva | grape |
| Artefacts (ART) | aereo | airplane | automobile | car |
|  | argento | silver | bicicletta | bicycle |
|  | calzino | sock | cappello | hat |
|  | canoa | canoe | cenere | ash |
|  | casco | helmet | cera | wax |
|  | cemento | cement | cravatta | tie |
|  | coltello | knife | diamante | diamond |
|  | cristallo | crystal | elicottero | helicopter |
|  | cucchiaio | spoon | falce | sickle |
|  | divano | couch | ferro | iron |
|  | forbici | scissors | forchetta | fork |
|  | lampada | bulb | ghiaccio | ice |
|  | martello | hammer | giacca | blazer |
|  | ombrello | umbrella | letto | bed |
|  | pantaloni | trousers | libreria | bookstore |
|  | perla | pearl | matita | pencil |
|  | pistola | gun | pennello | brush |
|  | polvere | dust | poltrona | armchair |
|  | scrivania | desk | sabbia | sand |
|  | sedia | chair | scarpa | shoe |
|  | stivale | boot | scopa | broom |
|  | trattore | tractor | specchio | mirror |
|  | uniforme | uniform | tavolo | table |
|  | veleno | poison | trapano | drill |

**Table 4.** Variables used in the selection of the experimental stimuli, and results of the independent samples t-tests comparing abstract and concrete nouns.

| **Concrete vs abstract nouns** | **t-test** | |
| --- | --- | --- |
| ***Variable*** | **t** | **p** |
| **Written frequency** ( from COLFIS database) | 1.637 | .103 |
| **Number order of the sense** (from Multi Word Net) | 0.451 | .652 |
| **Concreteness (CNC)** (from Della Rosa et al., 2010) | -65.879 | **< .001** |
| **Imageability (IMG)** (from Della Rosa et al., 2010) | -40.735 | **< .001** |
| **Abstractness (ABS)** (from Della Rosa et al., 2010) | -54.543 | **< .001** |
| **Context availability (CA)** (from Della Rosa et al., 2010) | -25.555 | **< .001** |
| **Age of acquisition (AoA)** (from Della Rosa et al., 2010) | -17.331 | **< .001** |
| **Mode of acquisition (MoA)** (from Della Rosa et al., 2010) | 23.148 | **< .001** |

**Experimental stimuli conditions and lists**

In the abstract domain, the Same Category condition included 48 pairs (i.e., 12 EM-EM, 12 ACT-ACT, 12 ATT-ATT, 12 COG-COG) and the Different Category condition included a total of 144 pairs (i.e. 36 X-EM, 36 X-ACT, 36 X-ATT, 36 X-COG) resulting from the combination of each target category with the other three remaining prime categories (i.e. 12 ATT-EM, 12 ACT-EM, 12 COG-EM; 12 EM-ACT, 12 ATT-ACT, 12 COG-ACT; 12 EM-ATT, 12 ACT-ATT, 12 COG-ATT; 12 EM-COG, 12 ACT-COG,12 ATT-COG). The category membership conditions were organized in six lists, each one presented to 6 participants, and divided into two experimental sessions in a counterbalanced way. Each list contained 24 word pairs of the Same Category condition and 24 word pairs of the Different Category condition. In order to avoid the repetition of the same word for each participant, within each list, the Same and Different conditions included word pairs belonging only to two categories, with categories never overlapping between the two conditions (i.e. SAME: 12 pairs EM-EM and 12 pairs COG-COG; DIFFERENT: 12 pairs ACT-ATT and 12 pairs ATT-ACT). The full rotation of combinations of categories in the SAME or DIFFERENT conditions is reported in Table 3.

In the concrete domain, the Same Category and Different Category conditions included 48 pairs each (i.e., Same Category: 24 BIOL-BIOL and 24 ART-ART; Different Category: 24 ART-BIOL and 24 BIOL-ART). Same and Different Category conditions in the concrete domain were organized in two pseudorandomised lists in the sense that if a prime preceded a target of the same category in one list, it appeared before a different category target in the other list (e.g., Italian: pera-GUFO, English: pear-OWL in list 1; Italian: pera-SCARPA; English: pear-SHOE in list 2). Each participant was presented with each word once. Each list contained 24 pairs of the Same Category (i.e., 12 BIOL-BIOL and 12 ART-ART) and 24 pairs of the Different Category condition (i.e., 12 ART-BIOL and 12 BIOL-ART). The two lists were presented to half of the participants (n=18, see Table 3 below) and each list was divided between two experimental sessions in a counterbalanced way.

**Table 5.** Lists with the full rotation of combinations of abstract and concrete categories.

| **Abstract concepts** | | | **Concrete concepts** |
| --- | --- | --- | --- |
| **List number** | **Same** | **Different** | **List number** |
| list 1 | COG-COG | ACT-ATT | list 2 |
|  | EM-EM | ATT-ACT |  |
| list 2 | ATT-ATT | EM-COG | list 1 |
|  | ACT-ACT | COG-EM |  |
| list 3 | ATT-ATT | ACT-COG | list 2 |
|  | EM-EM | COG-ACT |  |
| list 4 | ATT-ATT | EM-ACT | list 1 |
|  | COG-COG | ACT-EM |  |
| list 5 | ACT-ACT | EM-ATT | list 1 |
|  | COG-COG | ATT-EM |  |
| list 6 | ACT-ACT | ATT-COG | list 2 |
|  | EM-EM | COG-ATT |  |

We matched the 12 words used in the Same Word Condition (listed in Table 4 below) with both prime and target words belonging to the 6 categories in the Same Category condition for number of letters (prime: all p-values > .884; target: p-values= 1) and frequency (prime: all p-values= 1; target: p-values > .151), as in Wheatley et al., 2005.

**Table 6.** List of the stimuli used in the Same Word Condition.

| ***Domain*** | ***word (Italian)*** | ***word (English)*** |
| --- | --- | --- |
| Abstract | legge | law |
|  | ltupore | astonishment |
|  | lunzione | function |
|  | preferenza | preference |
|  | suggestione | suggestion |
|  | indifferenza | indifference |
| Concrete | sole | sun |
|  | latte | milk |
|  | barba | beard |
|  | scuola | school |
|  | pescatore | fisherman |
|  | labirinto | maze |

**Table 7.** List of the papers included for the selection of the regions of interest.

| **paper** | **type of study** | **technique** |
| --- | --- | --- |
| Beauregard, M., Chertkow, H., Bub, D., Murtha, S., Dixon, R., & Evans, A. (1997). The neural substrate for concrete, abstract, and emotional word lexica a positron emission tomography study. Journal of cognitive neuroscience, 9(4), 441-461. | original research | PET |
| Binney, R. J., Embleton, K. V., Jefferies, E., Parker, G. J., & Lambon Ralph, M. A. (2010). The ventral and inferolateral aspects of the anterior temporal lobe are crucial in semantic memory: evidence from a novel direct comparison of distortion-corrected fMRI, rTMS, and semantic dementia. Cerebral cortex, 20(11), 2728-2738. | original research | fMRI |
| Binney, R. J., Hoffman, P., Ralph, L., & Matthew, A. (2016). Mapping the multiple graded contributions of the anterior temporal lobe representational hub to abstract and social concepts: evidence from distortion-corrected fMRI. Cerebral Cortex, 26(11), 4227-4241. | original research | fMRI |
| Carter, C. S., Macdonald, A. M., Botvinick, M., Ross, L. L., Stenger, V. A., Noll, D., & Cohen, J. D. (2000). Parsing executive processes: strategic vs. evaluative functions of the anterior cingulate cortex. Proceedings of the National Academy of Sciences, 97(4), 1944-1948. | original research | fMRI |
| Casey, B. J., Thomas, K. M., Welsh, T. F., Badgaiyan, R. D., Eccard, C. H., Jennings, J. R., & Crone, E. A. (2000). Dissociation of response conflict, attentional selection, and expectancy with functional magnetic resonance imaging. Proceedings of the National Academy of Sciences, 97(15), 8728-8733. | original research | fMRI |
| Chao, L. L., & Martin, A. (2000). Representation of manipulable man-made objects in the dorsal stream. Neuroimage, 12(4), 478-484. | original research | fMRI |
| Chouinard, P. A., & Goodale, M. A. (2010). Category-specific neural processing for naming pictures of animals and naming pictures of tools: an ALE meta-analysis. Neuropsychologia, 48(2), 409-418. | meta-analysis | fMRI, PET |
| Clarke, A., & Tyler, L. K. (2014). Object-specific semantic coding in human perirhinal cortex. Journal of Neuroscience, 34(14), 4766-4775. | original research | fMRI |
| Costafreda, S. G., Brammer, M. J., David, A. S., & Fu, C. H. (2008). Predictors of amygdala activation during the processing of emotional stimuli: a meta-analysis of 385 PET and fMRI studies. Brain research reviews, 58(1), 57-70. | meta-analysis | fMRI, PET |
| Cotelli, M., Fertonani, A., Miozzo, A., Rosini, S., Manenti, R., Padovani, A., ... & Miniussi, C. (2011). Anomia training and brain stimulation in chronic aphasia. Neuropsychological rehabilitation, 21(5), 717-741. | original research | rTMS |
| Davey, J., Rueschemeyer, S. A., Costigan, A., Murphy, N., Krieger-Redwood, K., Hallam, G., & Jefferies, E. (2015). Shared neural processes support semantic control and action understanding. Brain and language, 142, 24-35. | original research | rTMS |
| Desai, R. H., Reilly, M., & van Dam, W. (2018). The multifaceted abstract brain. Phil. Trans. R. Soc. B, 373(1752), 20170122. | meta-analysis | fMRI |
| Durston, S., Davidson, M. C., Thomas, K. M., Worden, M. S., Tottenham, N., Martinez, A., ... & Casey, B. J. (2003). Parametric manipulation of conflict and response competition using rapid mixed-trial event-related fMRI. Neuroimage, 20(4), 2135-2141. | original research | fMRI |
| Egner, T., & Hirsch, J. (2005). The neural correlates and functional integration of cognitive control in a Stroop task. Neuroimage, 24(2), 539-547. | original research | fMRI |
| Gold, B. T., Balota, D. A., Kirchhoff, B. A., & Buckner, R. L. (2005). Common and dissociable activation patterns associated with controlled semantic and phonological processing: evidence from FMRI adaptation. Cerebral Cortex, 15(9), 1438-1450. | original research | fMRI |
| Hallam, G. P., Whitney, C., Hymers, M., Gouws, A. D., & Jefferies, E. (2016). Charting the effects of TMS with fMRI: Modulation of cortical recruitment within the distributed network supporting semantic control. Neuropsychologia, 93, 40-52. | original research | TMS-fMRI |
| Hoffman, P., Binney, R. J., & Ralph, M. A. L. (2015). Differing contributions of inferior prefrontal and anterior temporal cortex to concrete and abstract conceptual knowledge. Cortex, 63, 250-266. | original research | fMRI |
| Kensinger, E. A., & Schacter, D. L. (2006). Processing emotional pictures and words: Effects of valence and arousal. Cognitive, Affective, & Behavioral Neuroscience, 6(2), 110-126. | original research | fMRI |
| Kerns, J. G., Cohen, J. D., MacDonald, A. W., Cho, R. Y., Stenger, V. A., & Carter, C. S. (2004). Anterior cingulate conflict monitoring and adjustments in control. Science, 303(5660), 1023-1026. | original research | fMRI |
| Kerns, J. G. (2006). Anterior cingulate and prefrontal cortex activity in an FMRI study of trial-to-trial adjustments on the Simon task. Neuroimage, 33(1), 399-405. | original research | fMRI |
| Kim, H. (2017). Brain regions that show repetition suppression and enhancement: A meta‐analysis of 137 neuroimaging experiments. Human brain mapping, 38(4), 1894-1913. | meta-analysis | fMRI, PET |
| Kim, C., Johnson, N. F., & Gold, B. T. (2014). Conflict adaptation in prefrontal cortex: now you see it, now you don't. Cortex, 50, 76-85. | original research | fMRI |
| Lewis, P. A., Critchley, H. D., Rotshtein, P., & Dolan, R. J. (2007). Neural correlates of processing valence and arousal in affective words. Cerebral cortex, 17(3), 742-748. | original research | fMRI |
| Lungu, O. V., Binenstock, M. M., Pline, M. A., Yeaton, J. R., & Carey, J. R. (2007). Neural changes in control implementation of a continuous task. Journal of Neuroscience, 27(11), 3010-3016. | original research | fMRI |
| Moseley, R., Carota, F., Hauk, O., Mohr, B., & Pulvermüller, F. (2012). A role for the motor system in binding abstract emotional meaning. Cerebral cortex, 22(7), 1634-1647. | original research | fMRI |
| Noonan, K. A., Jefferies, E., Visser, M., & Lambon Ralph, M. A. (2013). Going beyond inferior prefrontal involvement in semantic control: evidence for the additional contribution of dorsal angular gyrus and posterior middle temporal cortex. Journal of cognitive neuroscience, 25(11), 1824-1850. | meta-analysis | fMRI, PET |
| Rice, G. E., Hoffman, P., Binney, R. J., & Lambon Ralph, M. A. (2018). Concrete versus abstract forms of social concept: an fMRI comparison of knowledge about people versus social terms. Philosophical Transactions of the Royal Society B: Biological Sciences, 373(1752), 20170136. | original research | fMRI |
| Ross, L. A., & Olson, I. R. (2010). Social cognition and the anterior temporal lobes. Neuroimage, 49(4), 3452-3462. | original research | fMRI |
| Skipper, L. M., & Olson, I. R. (2014). Semantic memory: Distinct neural representations for abstractness and valence. Brain and Language, 130, 1-10. | original research | fMRI |
| Taylor, K. I., Moss, H. E., Stamatakis, E. A., & Tyler, L. K. (2006). Binding crossmodal object features in perirhinal cortex. Proceedings of the National Academy of Sciences, 103(21), 8239-8244. | original research | fMRI |
| Van Veen, V., & Carter, C. S. (2005). Separating semantic conflict and response conflict in the Stroop task: a functional MRI study. Neuroimage, 27(3), 497-504. | original research | fMRI |
| Vigliocco, G., Kousta, S. T., Della Rosa, P. A., Vinson, D. P., Tettamanti, M., Devlin, J. T., & Cappa, S. F. (2013). The neural representation of abstract words: the role of emotion. Cerebral Cortex, 24(7), 1767-1777. | original research | fMRI |
| Vigneau, M., Beaucousin, V., Herve, P. Y., Duffau, H., Crivello, F., Houde, O., ... & Tzourio-Mazoyer, N. (2006). Meta-analyzing left hemisphere language areas: phonology, semantics, and sentence processing. Neuroimage, 30(4), 1414-1432. | meta-analysis | fMRI, PET |
| Visser, M., Embleton, K. V., Jefferies, E., Parker, G. J., & Ralph, M. L. (2010). The inferior, anterior temporal lobes and semantic memory clarified: novel evidence from distortion-corrected fMRI. Neuropsychologia, 48(6), 1689-1696. | original research | fMRI |
| Wang, J., Conder, J. A., Blitzer, D. N., & Shinkareva, S. V. (2010). Neural representation of abstract and concrete concepts: A meta‐analysis of neuroimaging studies. Human brain mapping, 31(10), 1459-1468. | meta-analysis | fMRI, PET |
| Whitney, C., Kirk, M., O'Sullivan, J., Lambon Ralph, M. A., & Jefferies, E. (2011). The neural organization of semantic control: TMS evidence for a distributed network in left inferior frontal and posterior middle temporal gyrus. Cerebral cortex, 21(5), 1066-1075. | original research | rTMS |
| Wilson-Mendenhall, C. D., Simmons, W. K., Martin, A., & Barsalou, L. W. (2013). Contextual processing of abstract concepts reveals neural representations of nonlinguistic semantic content. Journal of cognitive neuroscience, 25(6), 920-935. | original research | fMRI |
| Zahn, R., Moll, J., Krueger, F., Huey, E. D., Garrido, G., & Grafman, J. (2007). Social concepts are represented in the superior anterior temporal cortex. Proceedings of the National Academy of Sciences, 104(15), 6430-6435. | original research | fMRI |

**Table 8.** Characterization of the 15 semantic and 11 control regions by means of the Literature-Based (LB) approach (columns in orange), the information in the BrainMap (BM) databases (columns in blue), and the Semantic-Control differential measure (LB-BM columns in pink).

For example, consider the left anterior fusiform gyrus. According to LB information, it had a correction level index of 0.92, indicating that activity in this region mostly survived voxel level correction and a semantics/control sensitivity index value of 1, thus suggesting the region’s involvement in semantics. In BM database, the region, named ‘FuG_L_3_1’, is mainly involved in Cognition and Language, and had a control type mean of 1, indicting a high specificity in semantic paradigms. The region displayed a Semantic control differential value of 0, which indicated the concordance between LB and BM information.

|  | **LB Regions** | **Correction Level** | **Semantic/Control Sensitivity** | **BM Regions** | **Behavioural Domain** | | | **Control type mean** | **LB -BM** |
| --- | --- | --- | --- | --- | --- | --- | --- | --- | --- |
|  |  |  |  |  | **Label** | **P (activation\|domain)** | **Domain specificity** |  |  |
| **semantic regions** | L anterior fusiform gyrus | 0.92 | 1.00 | FuG_L_3_1 | Cognition.Language.Semantics  Cognition.Language.Speech  Cognition.Memory.Explicit | 3.08  1.98  1.95 | 1.51  0.97  0.80 | 1.00 | 0.00 |
|  | L posterior MTG | 0.33 | 0.13 | pSTS_L_2_2 | Cognition.Language.Speech  Cognition.Language.Orthography  Cognition.Language.Semantics  Cognition.Language.Syntax  Perception.Audition | 4.32  2.84  2.51  2.38  1.68 | 3.33  2.19  1.94  1.84  0.17 | 1.00 | -0.86 |
|  | L pars opercularis/ triangularis (BA44/45) | 0.43 | 0.06 | IFG_L_6_3 | Cognition.Memory.Explicit  Cognition.Language.Semantics  Cognition.Language.Syntax  Cognition.Language.Speech | 3.84  3.55  2.20  1.65 | 1.77  2.52  1.56  1.17 | 1.00 | -0.94 |
|  | L pars orbitalis (IFG, BA47) | -0.70 | 0.83 | OrG_L_6_6 | Emotion  Cognition.Language.Semantics  Cognition.Memory.Explicit | 1.90  1.46  1.44 | na  0.32  0.44 | 1.00 | -0.17 |
|  | R angular gyrus | 0.33 | 0.33 | IPL_R_6_1 | Cognition.SocialCognition  Perception.Vision.Shape  Perception.Vision.Motion  Cognition.Space | 2.55  2.29  2.18  2.07 | 0.13  1.37  1.31  0.11 | 1.00 | -0.67 |
|  | L middle frontal gyrus | 0.22 | -0.11 | MFG_L_7_1 | Action.Inhibition  Cognition.SocialCognition | 2.68  2.13 | 0.19  0.06 | 1.00 | -1.11 |
|  | L anterior hippocampus | 0.50 | 0.50 | Hipp_L_2_1 | Emotion.Sadness  Perception.Gustation  Emotion.Disgust  Emotion.Happiness  Emotion  Emotion.Fear  Perception.Olfaction  Cognition.Memory.Explicit | 4.88  4.49  3.51  3.32  3.17  2.75  2.34  1.50 | 2.11  0.30  1.51  1.16  na  1.19  0.16  0.40 | 0.83 | -0.33 |
|  | L angular gyrus | 0.17 | 0.00 | IPL_L_6_5 | Emotion  Cognition.Language  Cognition  Cognition.Memory.Explicit  Cognition.SocialCognition | 3.47  2.69  1.87  1.46  1.36 | na  0.73  na  0.52  0.14 | 0.83 | -0.83 |
|  | L posterior-lateral fusiform gyrus, L ITG | 0.50 | 0.89 | FuG_L_3_3 | Cognition.Language.Speech  Cognition.Language  Cognition.Language.Orthography  Cognition.Language.Phonology  Cognition.Language.Semantics  Perception.Vision.Shape | 2.89  2.56  2.56  2.24  2.06  1.51 | 2.79  2.47  2.47  2.16  1.99  0.45 | 0.80 | 0.09 |
|  | R lateral occipital area | 1.00 | 1.00 | OcG_R_4_4 | Perception.Vision.Shape  Perception.Vision  Cognition.Space  Cognition.Language.Orthography | 3.20  2.76  2.25  1.72 | 1.92  1.66  0.12  0.38 | 0.75 | 0.25 |
|  | R anterior fusiform gyrus | 1.00 | 1.00 | FuG_R_3_3 | Cognition.Language.Orthography  Emotion.Anger  Interoception.Sexuality  Cognition.Language  Action.Observation  Cognition.Space  Perception.Vision  Cognition.Language.Semantics  Emotion.Fear  Perception.Vision.Shape | 3.91  3.57  2.75  2.32  2.23  2.11  1.94  1.93  1.83  1.58 | 2.12  0.88  0.05  1.26  0.06  0.09  1.05  1.05  0.45  0.85 | 0.75 | 0.25 |
|  | R posterior-medial fusiform gyrus | 0.00 | 0.75 | FuG_R_3_2 | Perception.Vision  Cognition.Language  Emotion.Fear  Perception.Vision.Shape  Action.MotorLearning | 3.86  2.82  2.47  2.46  1.86 | 2.12  0.51  0.31  1.35  0.07 | 0.75 | 0.00 |
|  | L middle ATL | -0.33 | 0.83 | MTG_L_4_2 | Cognition.SocialCognition  Cognition.Language | 10.97  4.92 | 1.15  1.34 | 0.67 | 0.16 |
|  | R precuneus | 0.00 | 0.60 | Pcun_R_4_4 | Cognition.SocialCognition  Cognition.Memory.Explicit | 3.11  2.45 | 0.33  0.87 | 0.67 | -0.07 |
|  | L superior frontal gyrus | -0.10 | 0.10 | SFG_L_7_3 | Cognition.Memory.Explicit  Emotion  Cognition.SocialCognition | 4.97  2.24  2.14 | 1.50  na  0.11 | 0.67 | -0.57 |
| **control regions** | L dorsolateral PFC | 0.17 | 0.00 | MFG_L_7_2 | Cognition.Language.Phonology  Cognition.Language.Semantics  Cognition.Memory.Explicit  Cognition.Memory.Working | 1.82  1.80  1.63  1.45 | 0.99  0.98  1.16  1.03 | -0.60 | 0.60 |
|  | R posterior superior temporal gyrus | 1.00 | 1.00 | IPL_R_6_6 | Action.Execution  Perception.Somesthesis  Perception.Somesthesis.Pain | 3.89  3.27  2.05 | 2.22  3.60  2.26 | -1.00 | 2.00 |
|  | L supramarginal gyrus | 0.00 | 0.42 | IPL_L_6_6 | Action.Execution  Perception.Audition  Perception.Somesthesis  Perception.Somesthesis.Pain | 3.20  2.63  1.68  2.18 | 1.83  0.39  1.93  2.51 | -1.00 | 1.42 |
|  | R pars opercularis/ triangularis (BA44/45) | 0.33 | 1.00 | IFG_R_6_1 | Cognition.Reasoning  Cognition.Attention | 2.35  1.52 | 0.25  0.16 | -1.00 | 2.00 |
|  | R caudate nuclues | 0.50 | 1.00 | Str_R_6_1 | Cognition  Perception.Gustation  Emotion | 3,36  2,86  2,51 | na  0,10  na | -1.00 | 2.00 |
|  | L dorsomedial PFC | 0.75 | 0.50 | SFG_L_7_6 | Cognition.Memory.Explicit | 1.71 | 0.52 | -1.00 | 1.50 |
|  | anterior cingulate cortex | 0.11 | -0.22 | CG_L_7_3 | Perception.Gustation  Emotion.Sadness  Perception.Somesthesis.Pain  Cognition  Emotion | 2.95  2.45  2.19  1.86  1.83 | 0.20  0.37  1.24  na  na | -1.00 | 0.78 |
|  | R pars triangularis (IFG, BA45) | 0.00 | -1.00 | IFG_R_6_5 | Cognition.Time  Perception.Somesthesis.Pain | 3.28  2.14 | 0.09  1.18 | -1.00 | 0.00 |
|  | R supramarginal gyrus | 1.00 | -1.00 | IPL_R_6_4 | Interoception.Bladder  Cognition.Attention  Perception.Vision.Motion  Action.Execution  Perception.Somesthesis.Pain  Action.Inhibition | 2.99  2.46  2.10  1.98  1.43  1.37 | na  0.03  0.63  1.13  0.86  0.10 | -1.00 | 0.00 |
|  | L dorsal angular gyrus | 1.00 | -1.00 | IPL_L_6_2 | Cognition.Space  Cognition.Reasoning  Cognition.Memory.Working | 2.29  2.06  1.88 | 0.36  0.33  0.77 | -1.00 | 0.00 |
|  | L caudate nuclues | -1.00 | -1.00 | Str_L_6_5 | Cognition  Emotion | 1.91  1.78 | na  na | -1.00 | 0.00 |

**Outliers screening**

Outliers screening was divided into two steps.

First, we calculated the mean BOLD signal in beta images (n= 20: 10 beta images x 2 sessions) in each ROI (n= 26) (columns) for each subject (n= 36) (rows), thus obtaining a subjects (row) (n=36) X region (columns) (n= 26) matrix including BOLD signal estimates extracted for all betas relative to all conditions of interest (n= 20 x subject).

Second, we calculated 1) the mean (e.g., mean Beta s1) and standard deviation (e.g., st.dev. Beta s1) for each subject across all the ROIs; 2) the mean and standard deviation for each ROI across all subjects. Third we calculated the global mean and standard deviation relative to 1) all subjects (n= 36) and 2) ROIs (n= 26). Subjects were excluded if beta values were above or below 3 standard deviations from the global mean in more than 1/3 of the ROIs (n> 9). ROIs were excluded if beta values were above or below 3 standard deviations from the global mean in more than 1/3 of the subjects (n> 12).

See Figure 1 below.

**Figure 1.** Illustration of the outliers screening procedure. The matrix represents the mean BOLD signal in the 20 beta images in each Region of Interest (n= 26) (columns) for each subject (n= 36) (rows).

Highlighted in red: the mean and standard deviation for each subject across the Regions of Interest (e.g. mean Beta subj.01 and st.dev. Beta subj. 01), the mean and standard deviation for each Region of Interest across all subjects (e.g. mean Beta ROI 01 and st.dev. Beta ROI 01), and the corresponding global means and standard deviations. See text for the criteria of exclusion of subjects and Region of Interests and for additional details. Subj.= subject; ROI= Region of Interest; β= beta; st.dev.= standard deviation.


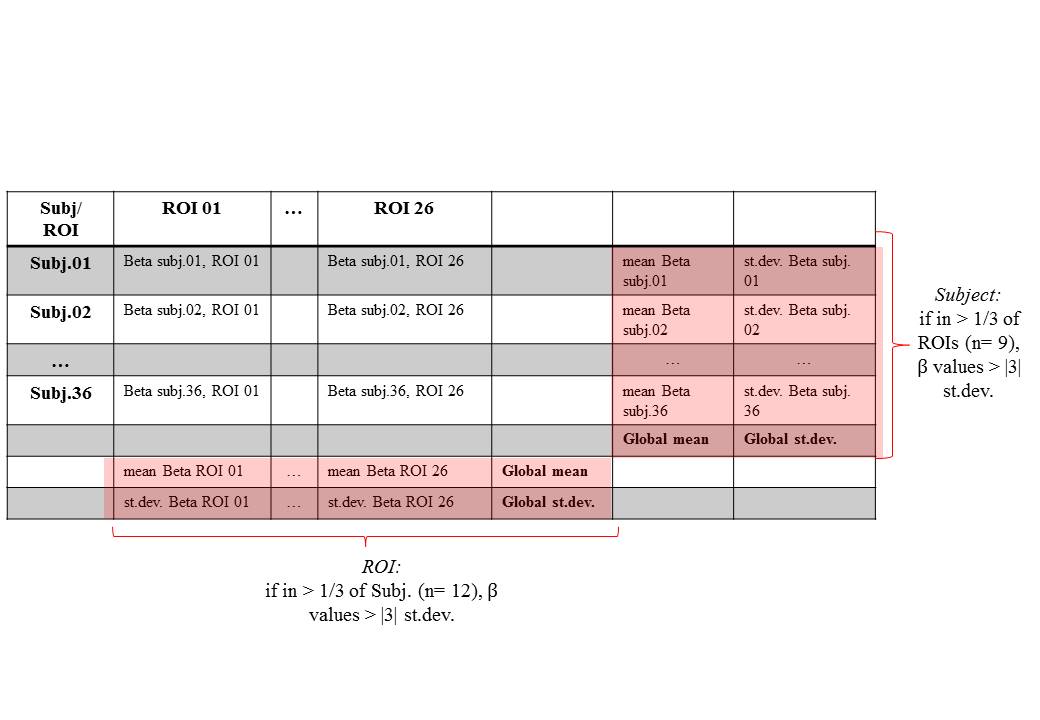

Supplement: Supplementary file 1 — Supplementary Information. [file 41598_2021_2013_MOESM1_ESM.docx]
